# Preclinical evaluation of a candidate naked plasmid DNA vaccine against SARS-CoV-2

**DOI:** 10.1038/s41541-021-00419-z

**Published:** 2021-12-20

**Authors:** Ria Lassaunière, Charlotta Polacek, Gregers J. Gram, Anders Frische, Jeanette Linnea Tingstedt, Maren Krüger, Brigitte G. Dorner, Anthony Cook, Renita Brown, Tatyana Orekov, Tammy Putmon-Taylor, Tracey-Ann Campbell, Jack Greenhouse, Laurent Pessaint, Hanne Andersen, Mark G. Lewis, Anders Fomsgaard

**Affiliations:** 1 Department of Virus and Microbiological Special Diagnostic, Statens Serum Institut, Copenhagen, Denmark; 2 Department of Business Support and Campus, Statens Serum Institut, Copenhagen, Denmark; 3 Biological Toxins (ZBS 3), Centre for Biological Threats and Special Pathogens, Robert Koch Institute, Berlin, Germany; 4 BIOQUAL Inc., Rockville, DM 20850 USA; 5 Infectious Disease Research Unit, Clinical Institute, University of Southern Denmark, Odense, Denmark

**Keywords:** DNA vaccines, SARS-CoV-2, DNA vaccines

## Abstract

New generation plasmid DNA vaccines may be a safe, fast and simple emergency vaccine platform for preparedness against emerging viral pathogens. Applying platform optimization strategies, we tested the pre-clinical immunogenicity and protective effect of a candidate DNA plasmid vaccine specific for severe acute respiratory syndrome coronavirus 2 (SARS-CoV-2). The DNA vaccine induced spike-specific binding IgG and neutralizing antibodies in mice, rabbits, and rhesus macaques together with robust Th1 dominant cellular responses in small animals. Intradermal and intramuscular needle-free administration of the DNA vaccine yielded comparable immune responses. In a vaccination-challenge study of rhesus macaques, the vaccine demonstrated protection from viral replication in the lungs following intranasal and intratracheal inoculation with SARS-CoV-2. In conclusion, the candidate plasmid DNA vaccine encoding the SARS-CoV-2 spike protein is immunogenic in different models and confers protection against lung infection in nonhuman primates. Further evaluation of this DNA vaccine candidate in clinical trials is warranted.

## Introduction

Coronavirus disease 2019 (COVID-19), caused by severe acute respiratory syndrome coronavirus 2 (SARS-CoV-2), emerged in Wuhan, China around December 2019. It has since caused a global pandemic that has to date, resulted in over 160 million confirmed infections and 3.5 million deaths (WHO COVID-19 Weekly Epidemiological Update; 1 June 2021), although the numbers are likely underestimated^1^. Developing safe and effective vaccines and testing new vaccine platforms to control the pandemic are paramount.

The simplicity and stability of plasmid DNA vaccines make it an attractive immunization platform for emerging viral threats. The DNA vaccines can be designed and produced quickly once the genetic sequence is known and adapted rapidly to new emerging viral variants of concern. Clinically, the DNA vaccine modality is generally regarded as safe and is immunogenic in many different mammalian species including man^2,3^. Inducing both broad antibody and cellular immune responses, DNA vaccines have the potential to reduce both infection and disease. Intrinsically, the DNA vaccines are stable and can be freeze-dried, allowing for long-term storage at ambient temperature^4^. The plasmid DNA does not induce vector-specific antibodies, thus permitting multiple booster vaccinations including mixed modality prime-boost strategies^5^.

Historically, first-generation DNA vaccines performed poorly in primates. This was compounded by the application of the platform to complex pathogens where the correlates of protection are undefined and where other traditional vaccines have similarly failed, such as human immunodeficiency virus (HIV). However, continued platform optimization has seen to the improved performance of DNA vaccines in nonhuman primates and man. For example, a candidate Zika virus DNA vaccine protected rhesus macaques against viremia following Zika virus challenge and induced neutralizing antibody titers >300 when delivered intramuscularly with the needle-free Stratis Device (PharmaJet®)^6,7^. A different flavivirus DNA vaccine, targeting West Nile virus, is FDA approved for horses but also induced T cell and neutralizing antibody responses in humans^8^. Furthermore, an influenza trivalent DNA vaccine conferred protection against influenza challenge in a phase 1b clinical trial^9^ and, through cell-mediated immunity, a human papillomavirus DNA vaccine aided the regression of lesions and viral clearance in cervical intraepithelial neoplasia-3 patients^10^.

Considering the success of mRNA vaccines and DNA delivered by recombinant viruses and the intrinsic advantages and recent improved performances of plasmid DNA vaccines, an evaluation of the plasmid DNA platform is warranted in the ongoing SARS-CoV-2 pandemic. Here we describe the pre-clinical evaluation of a candidate DNA vaccine that targets the spike protein of SARS-CoV-2. Using platform optimization strategies to improve safety, antigen expression, potency, and immunogenicity, we address shortcomings associated with first-generation DNA vaccines. These optimization strategies include using: (i) a vector that lacks any antibiotic resistance genes^11^; (ii) an optimally reduced size vector^12^; (iii) vaccine antigen codon optimization^13^; (iv) co-expression of an immune stimulatory Retinoic-acid-inducible gene I (RIG-I) agonist that facilitates a type 1 interferon response^14^; (v) high yield antibiotic-free production in a current Good Manufacturing Practice process^11,15^; and (vi) needle-free jet administration to the skin or muscle^16^.

Multiple SARS-CoV-2 vaccines are being developed at an unprecedented speed. To ensure thorough evaluation of the safety risks, potential autoimmune or hyper-immune reactions, and enhanced infection and/or disease, for as many different platforms as possible, all vaccines need to be thoroughly assessed for safety and immunogenicity and protection from viral challenge in animal models prior to clinical evaluation. Here we describe the evaluation of the immunogenicity of an optimized DNA plasmid vaccine candidate in mice, rabbits, and nonhuman primates, as well as an assessment of the protective effect in rhesus macaques, the most extensively used model for evaluation of SARS-CoV-2/COVID-19 vaccine protection.

## Results

### The DNA vaccine candidate

**Fig. 1:**
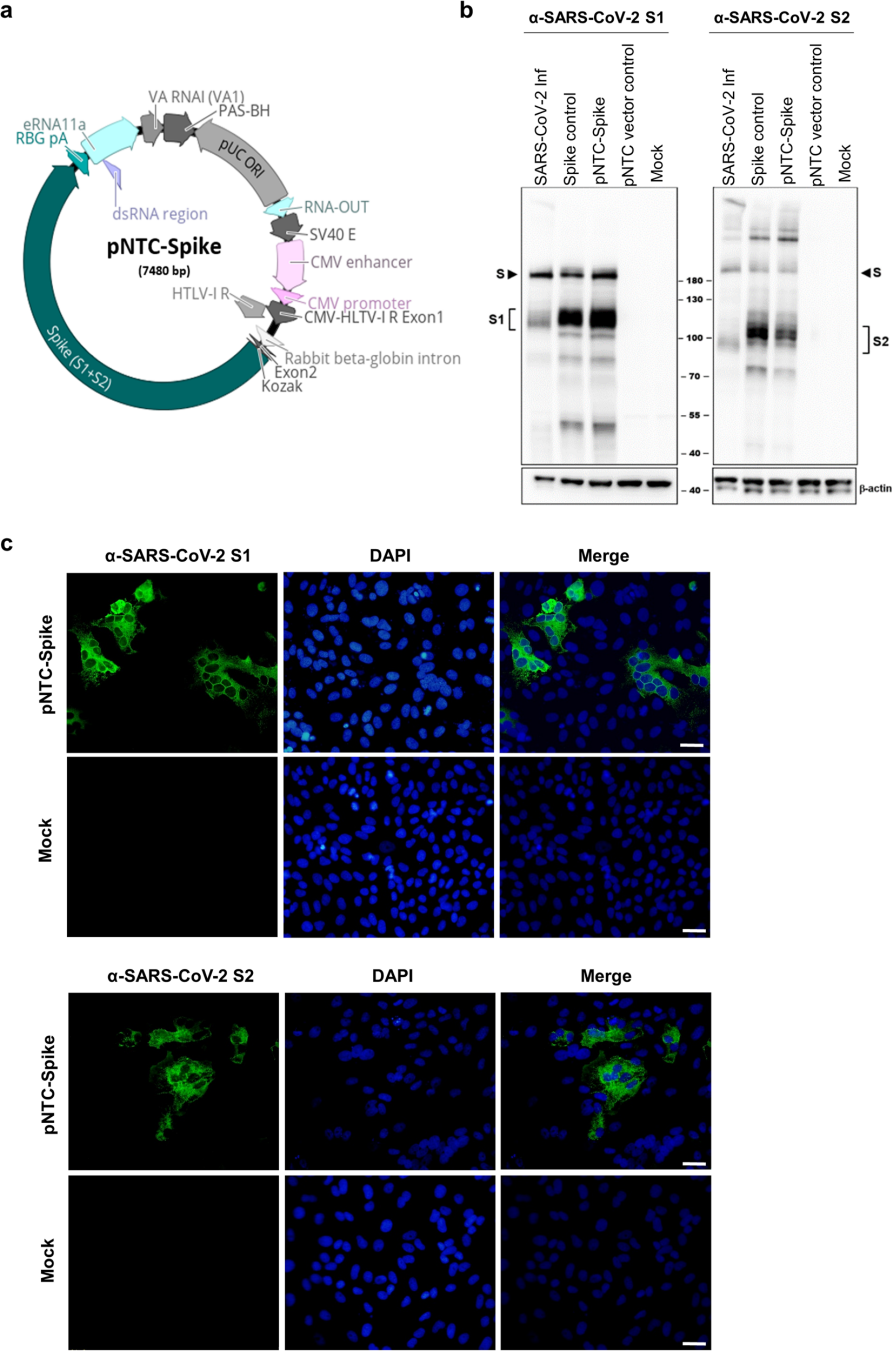
SARS-CoV-2 DNA vaccine. **a** DNA vaccine construct pNTC-Spike; full length, human codon optimized SARS-CoV-2 spike sequence cloned into expression vector NTC8685-eRNA41H with significant vector features indicated. **b** Protein expression from the DNA vaccine candidate was confirmed by DNA lipofection into Vero E6 cells followed by western blotting using anti-S1 (left) and anti-S2 (right) detecting mouse monoclonal antibodies S1-1047 and S2-1254, respectively. Lanes: (1) SARS-CoV-2 infected Vero E6 extract (2) Spike expressing plasmid (commercial positive control), (3) pNTC-Spike, (4) NTC vector control, and (5) VERO E6 (negative control). Lower panels show the 42 kDa β-actin expression as loading control. Full-length Spike protein and Spike S1/S2-fragments are indicated with solid arrows and brackets, respectively. All blots derived from the same experiment and were processed in parallel. Raw, uncropped blots are presented in Supplementary Fig. 2. **c** Expression of pNTC-Spike-derived SARS-CoV-2 spike protein in Vero E6 cells, visualized by immunofluorescent labeling. Forty-eight hours after transfection, cells were incubated with either the anti-S1 mouse monoclonal antibody S1-1047 (top panel) or anti-S2 mouse monoclonal antibody S2-1254 (bottom panel) followed by a goat anti-mouse IgG Alexa Fluor 488 conjugate for detection (green). Cell nuclei were counterstained with DAPI (blue). Size scale bar: 20 µm.

The DNA vaccine candidate hereafter referred to as pNTC-Spike, expresses an unmodified, wild-type full-length SARS-CoV-2 spike protein derived from the Wuhan-hu-1 reference strain. The human codon optimized nucleotide sequence was subcloned into the NTC8685-eRNA41H (Fig. 1a), a nano-plasmid eukaryotic expression vector approved for clinical use^17^. Considerations for the vector design are described in detail elsewhere^12^. Notable features of this vector backbone include: (i) the lack of any antibiotic resistance genes to improve DNA vaccine safety^15^; (ii) improved transgene expression over vectors containing antibiotic resistance genes^18^; (iii) co-expression of a RIG-I agonist that facilitates a type 1 interferon response and increases DNA vaccine-induced antibody and cellular responses^14^; and (iv) a reduced size (3.6 kb). NTC8685-eRNA41H has an established toxicity and bio-distribution profile and a documented history of testing in humans with other vaccine-gene inserts^17^. In order to demonstrate expression of the SARS-CoV-2 spike protein encoded by this optimized vector, we generated highly specific monoclonal antibodies (mAb) targeting the S1- or S2-domain of SARS-CoV-2 using hybridoma technology. Based on a stringent selection procedure, mAbs S1-1047 and S2-1254 were obtained that showed an excellent specificity against their respective target domain and no cross-reactivity against the related spike proteins of SARS-CoV, MERS-CoV or any of the four Coronaviruses pathogenic to humans (Supplementary Fig. 1).

Using these mAbs, Western blot analysis of Vero E6 cells transfected with pNTC-Spike confirmed the expression of a full-length spike protein of ~190 kDa with an intact cleavage site, as suggested by the concurrent detection of the S1 and S2 subunits at ~110 kDa and S2 at ~100 kDa (Fig. 1b). The spike protein fragment sizes correspond to that observed by others and reflect the glycosylation of the full-length spike protein and its S1 and S2 subunits with all fragments migrating higher than the predicted 141 kDa, 77 kDa/, and 65 kDa, respectively^19^. Additional fragments of <100 kDa may be cleavage products produced by other cellular proteases such as cathepsin B and L, elastase, and trypsin, which have been demonstrated to cleave coronavirus spike proteins^20,21^, although this warrants further characterization. The cellular distribution of expressed proteins from pNTC-Spike was visualized with immunofluorescence staining of Vero E6 cells 48 h post-transfection (Fig. 1c). SARS-CoV-2 spike S1 and S2 labeling with mAbs S1-1047 and S2-1254, respectively, showed cytoplasmic distribution for both proteins as reported^22^.

### Immunogenicity in mice

**Fig. 2:**
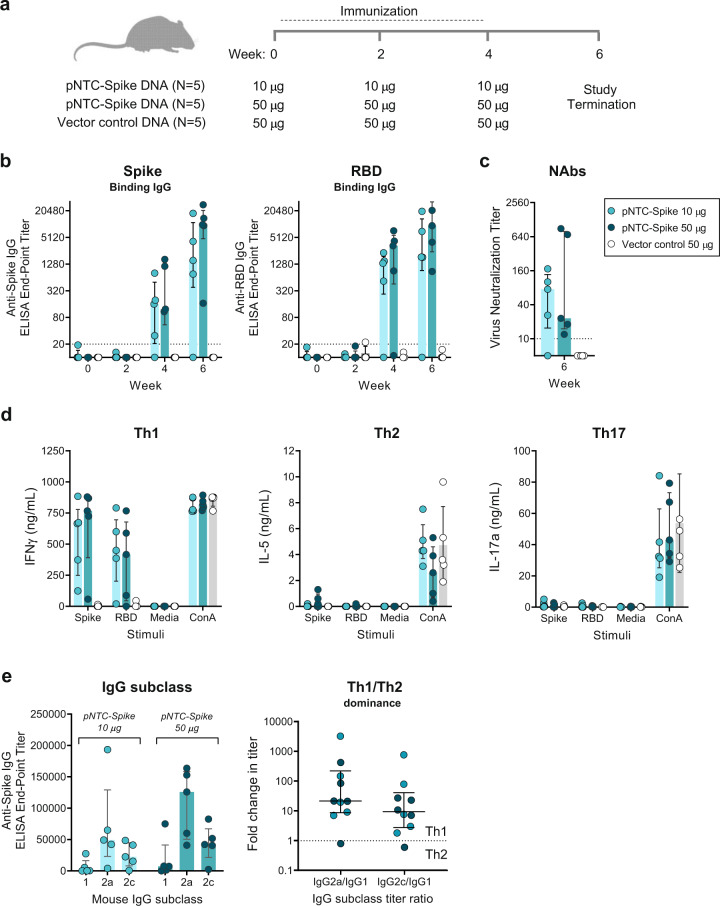
Humoral and cellular immune responses to pNTC-Spike in mice. **a** An overview of the immunization strategy in CB6F1 mice. Animals received three immunization of either 10 µg pNTC-Spike (*N* = 5), 50 µg pNTC-Spike (*N* = 5), or 50 µg vector control (*N* = 5) at 2 week intervals via the intradermal route using needle-and-syringe injection. **b** Temporal end-point titers of IgG antibodies specific for the SARS-CoV-2 spike ectodomain and receptor binding domain (RBD). Dotted lines indicate the assay limit of quantitation. **c** 50% virus-neutralizing antibody (NAb) titer against a SARS-CoV-2 clinical isolate as determined in a live virus microneutralization assay. Dotted lines indicate the assay limit of quantitation. **d** Interferon gamma (IFNγ), interleukin 5 (IL-5), and interleukin 17a (IL-17a) levels—representing T helper (Th) 1, Th2, and Th17 responses—measured by ELISA following restimulation of immunized rabbit splenocytes with the SARS-CoV-2 spike ectodomain and RBD. Cell culture medium and concanavalin A (ConA) served as antigen negative and positive controls, respectively. **e** End-point titers of SARS-CoV-2 spike-specific IgG subclasses in mice after the third immunization with either 10 µg or 50 µg pNTC-Spike (week 6). The Th1/Th2 dominance determined as the ratio of IgG2a:IgG1 and IgG2c:IgG1 end-point titers. Bar graphs indicate the median with interquartile range. Mouse silhouette created with BioRender.com.

The immunogenicity of the candidate DNA vaccine, pNTC-Spike, was first assessed in CB6F1 mice. These filial generation hybrid mice are a cross between C57BL/6 males and BALB/c females. With the parental strains each biased towards Th1 and Th2 responses, respectively, the cross confers a balanced Th1/Th2 responsiveness in the CB6F1 mice. The animals were immunized with either pNTC-Spike, in 10 µg (*N* = 5) or 50 µg (*N* = 5) doses, or vector control in a 50 µg dose (*N* = 5) without adjuvant at weeks 0, 2, and 4 by intradermal injection (Fig. 2a). Both 10 µg and 50 µg doses of pNTC-Spike elicited IgG responses specific to the SARS-CoV-2 spike ectodomain and receptor-binding domain (RBD) after the second immunization (week 4), which were further boosted by a subsequent immunization in a dose-dependent manner (week 6) (Fig. 2b). At week 6, the median end-point titer of antibodies specific for the spike ectodomain were 1547 (range: 10–17906) and 9887 (range: 167–27922) for the 10 µg and 50 µg group, respectively. All animals that developed spike- and RBD-specific binding IgG by ELISA also developed neutralizing antibodies as determined at week 6 in a SARS-CoV-2 virus microneutralization assay (Fig. 2c). Neutralizing antibody titers directly correlated with spike binding antibody titers (Spearman *r* = 0.758, *P* = 0.015) and RBD binding antibody titers (Spearman *r* = 0.818, *P* = 0.006). The limited volume of serum obtained from animals after the first and second immunization precluded an evaluation of neutralizing antibody responses at week 2 and week 4.

For mice immunized with pNTC-Spike, restimulation of splenocytes with the spike and RBD protein induced high levels interferon gamma (IFN-γ), indicative of a T helper (Th) 1 response, and comparatively low levels of interleukin (IL)−5 and IL-17 that represent Th2 and Th17 responses, respectively (Fig. 2c). To confirm the observed Th1 dominance, we measured SARS-CoV-2 spike-specific IgG subclass responses since the proportions of the different subclasses are dictated by the prevailing cytokine environment. IgG2a and IgG2c, which increase in response to the Th1 cytokine IFN-γ, occurred at notably higher titers relative to IgG1, which increases in response to the Th2 cytokine IL-4 (Fig. 2e). Overall, the ratio of IgG2a:IgG1 was 21.3 and the IgG2c:IgG1 ratio 9.2; thus, confirming the Th1 dominant vaccine response induced by the pNTC-Spike.

### Immunogenicity and safety in rabbits

**Fig. 3:**
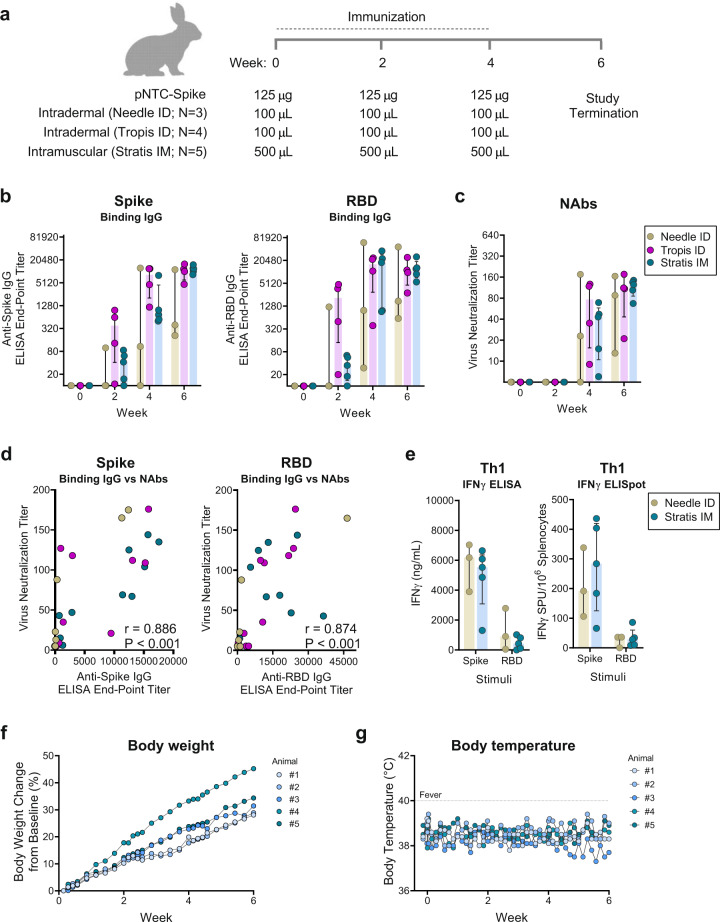
Humoral and cellular immune responses to pNTC-spike vaccination in rabbits. **a** An overview of the immunization strategy in New Zealand white rabbits. Animals received three immunization of 125 µg pNTC-Spike at 2 week intervals delivered either as a 100 µL intradermal dose using a needle-and-syringe (*N* = 3) or the needle-free PharmaJet® Tropis ID device (*N* = 4) or as a 500 µL intramuscular dose using the needle-free PharmaJet® Stratis IM device (*N* = 5). **b** Temporal end-point titers of IgG antibodies specific for the SARS-CoV-2 spike ectodomain and receptor-binding domain (RBD). **c** 50% virus-neutralizing antibody titer against a SARS-CoV-2 clinical isolate as determined in a live virus microneutralization assay. **d** Correlation analysis between the level of SARS-CoV-2 spike-specific binding IgG and virus neutralization. **e** Interferon gamma (IFNγ) responses measured by ELISA and ELISpot following restimulation of immunized rabbit splenocytes with the SARS-CoV-2 spike ectodomain and RBD. Cell culture medium and concanavalin A (ConA) served as antigen negative and positive controls, respectively. **f** Percentage body weight change from baseline (day 0) of animals measured daily for 4 days after each intramuscular immunization and every 2 day thereafter. **g** Body temperature measured on the day of each intramuscular immunization (0 and 6 h) and daily thereafter. A temperature above 40 °C constitutes a fever (dotted line). Bar graphs indicate the median with interquartile range. Rabbit silhouette created with BioRender.com.

The small size of mice precludes the evaluation of needle-free jet administration in this animal model. Therefore, to determine the effect of needle-free administration of the candidate DNA vaccine, we evaluated the immunogenicity and safety of pNTC-Spike in rabbits using either needle-and-syringe injection or needle-free jet-injection administrations to skin or muscle. New Zealand white rabbits (10 weeks old) received three immunizations of 125 µg DNA without adjuvant at weeks 0, 2, and 4 by the intradermal route using needle-and-syringe injection intradermal (*N* = 3) or the PharmaJet® Tropis ID device (*N* = 4) or via the intramuscular route using the PharmaJet® Stratis IM device (*N* = 5; Fig. 3a) in separate experiments. While calibrated for human use, the devices deliver liquid to the skin (Tropis ID) and muscle (Stratis IM) of rabbits as determined empirically using a liquid dye (Supplementary Fig. 3). Overall, needle-free administration of the vaccine via the intradermal and intramuscular route induced comparable binding IgG and neutralizing antibody responses that were higher and more consistent than intradermal injection using needle and syringe (Figs. 3b and 3c).

The pNTC-Spike vaccine-induced IgG responses specific to the spike ectodomain and RBD after the first immunization (week 2) and were boosted by subsequent immunizations (Fig. 3b). At week 6, the median spike ectodomain-specific end-point titer were 398 (range: 209–11349), 10975 (range: 4667–15880), and 11956 (range: 8349–15242) for the groups immunized using needle and syringe, needle-free Tropis ID, and needle-free Stratis IM, respectively. Virus-specific neutralizing antibodies were detected after the second immunization and increased following the third immunization reaching median 50% neutralization titers of 88 (range: 13–165), 111 (21–176), and 125 (67–144) at week 6 in the aforementioned groups, respectively (Fig. 3c). Neutralizing antibody titers directly correlated with spike binding antibody titers (Spearman *r* = 0.886, *P* < 0.001; Fig. 3d) and RBD binding antibody titers (Spearman *r* = 0.874, *P* < 0.001). For animals immunized intramuscularly and by needle-and-syringe, T cell induced immunity was also evaluated. Data for the Tropis ID group are unavailable. Restimulation of splenocytes with the spike and RBD protein induced IFN-γ responses as measured by both IFN-γ ELISPOT and IFN-γ cytokine ELISA (Fig. 3e). In these animals, the smaller RBD protein (234 amino acids) induced a lower IFN-γ response compared to the full-length spike protein (1209 amino acids). In contrast to that observed for antibody responses, the IFN-γ responses were comparable for animals immunized with intradermal needle-and-syringe administration and intramuscular needle-free jet administration.

Vaccine safety was observed in the rabbits that were immunized intramuscularly by monitoring temperature, body weight, clinical signs, and behavior. No cases of death, impending death, or obvious clinical signs were observed in any of the animals. Local site reactions were rare to absent. Only one animal developed slight swelling at the injection site 2 days after the second immunization that resolved 4 days later. All animals continued to gain weight throughout the study, and none suffered acute weight loss following any of the immunizations (Fig. 3f). None of the animals developed a fever (≥40 °C) on the day of immunization or in the days thereafter (Fig. 3g). The studies that evaluated intradermal administration of pNTC-Spike were not specifically designed to monitor safety. Nonetheless, there were no cases of death or impending death. Based on observations during standard care of the animals, there were no obvious clinical signs, and immunizations were well tolerated.

### Immunogenicity in rhesus macaques

**Fig. 4:**
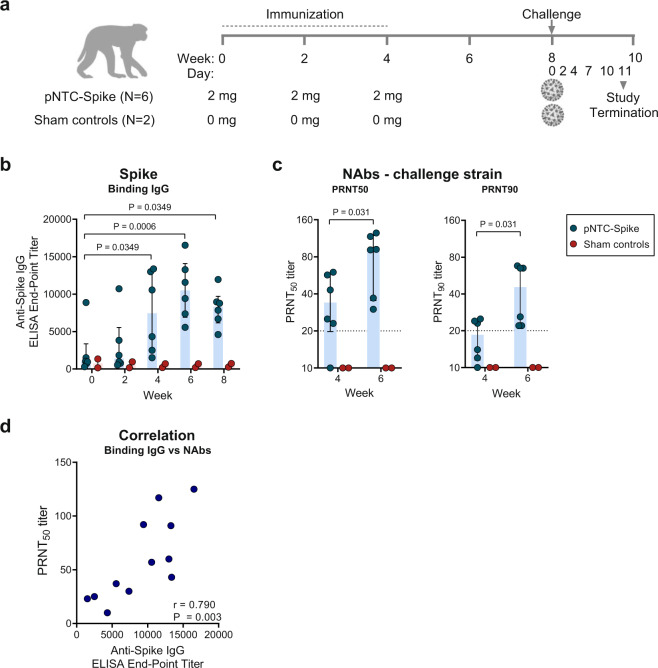
Humoral immune responses to pNTC-Spike in nonhuman primates. **a** An overview of immunogenicity and virus challenge study in rhesus macaques. pNTC-Spike vaccinates (*N* = 6) received three immunizations of 2 mg at 2 week intervals and were challenged with 1.0 × 10^5^ TCID_50_ (1.2 × 10^8^ RNA copies, 1.1 × 10^4^ PFU) SARS-CoV-2 (strain nCoV-WAI-2020; MN985325.1) 4 weeks after the final immunization (week 8). Sham controls (*N* = 2) were SARS-CoV-2 immune naïve until inoculation with the same SARS-CoV-2 viral dose at week 8. **b** End-point titers of binding IgG antibodies specific for the SARS-CoV-2 spike ectodomain. **c** Neutralizing antibody titers measures as a 50% inhibition of virus infection (PRTN_50_) and the more stringent 90% inhibition (PRNT_90_). **d** Correlation between anti-spike IgG binding antibody titers and 50% neutralization titers (PRNT_50_) for the challenge strain. Bar graphs indicate the median and interquartile range. Dotted lines indicate the assay limit of quantitation. SARS-CoV-2 virion image created with BioRender.com.

We next evaluated pNTC-Spike immunogenicity and protection against SARS-CoV-2 infection in a nonhuman primate model. Rhesus macaques (2–8 years old) were immunized with pNTC-Spike (*N* = 6) or were untreated sham controls (*N* = 2). The animals received three immunizations of 2 mg DNA without adjuvant at weeks 0, 2, and 4 by the intradermal route using the needle-free Tropis ID device (Fig. 4a). The immunizations were well tolerated based on daily cage-side observations of the animals during the study period. No adverse reactions at the injection sites were observed.

The candidate vaccine induced an increase in spike-specific binding IgG antibodies from baseline in all vaccinated animals after the second immunization (week 4; median end-point titer = 7440, range: 1509–13371) that were boosted by a third immunization (week 6; median end-point titer = 10494, range: 5569–16252) (Fig. 4b). One of the vaccinated animals had binding antibodies by ELISA at week 0. We speculate this might reflect the cross-reactivity of other natural primate coronaviruses. The pNTC-Spike vaccine nonetheless increased SARS-CoV-2-specific binding antibodies by ELISA in this animal. The ability of vaccine elicited antibodies to neutralize virus infection was evaluated with a live virus plaque reduction neutralization test (PRNT). Neutralizing antibodies capable of reducing plaque-forming units (PFU) by more than 50% at a serum dilution ≥1:20, were observed in five out of six vaccinated animals after the second immunization (median: PRNT_50_ = 34, range:10–60), and a 90% reduction (PRNT_90_) in three out of six vaccinated animals (Fig. 4c). Neutralizing antibody responses were boosted by the third immunization, with all vaccinated animals having developed neutralizing antibodies by week 6 measured as PRNT_50_ (median: PRNT_50_ = 92, range: 30–125) and the more stringent PRNT_90_ (median: PRNT_90_ = 46, range: 22–68), 2 weeks before virus challenge. Overall, the spike-specific binding IgG titers correlated with the PRTN_50_ titers (Spearman *r* = 0.790, *P* = 0.003; Fig. 4d).

### Protective effects of pNTC-Spike plasmid vaccine

**Fig. 5:**
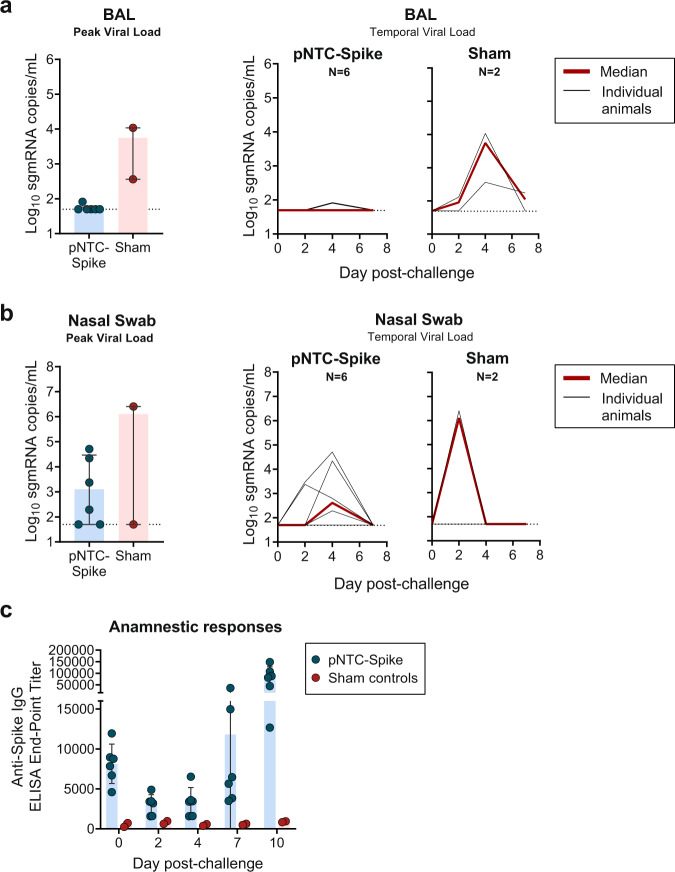
Viral load and anamnestic responses in rhesus macaques following challenge with SARS-CoV-2 by the intranasal and intratracheal route. **a** Peak and temporal SARS-CoV-2 viral loads, measured as SARS-CoV-2 E gene subgenomic mRNA, in BAL of pNTC-Spike vaccinated animals (*N* = 6) and untreated sham controls (*N* = 2). **b** Peak and temporal viral loads in nasal swabs of pNTC-Spike vaccinated animals and untreated sham controls. **c** End-point titers of binding IgG antibodies specific for the SARS-CoV-2 spike ectodomain on the day of virus challenge and days 2, 4, 7, and 10 following inoculation. Bar graphs indicate the median with interquartile range; Line graphs present each animal as a separate black line and the red line the median for each time point. Dotted lines indicate the assay limit of quantitation (1.69 log_10_ sgmRNA copies/mL).

At week 8, 4 weeks after the final immunization, all animals were challenged with 1.0 × 10^5^ TCID_50_ SARS-CoV-2 by the intranasal and intratracheal routes. The SARS-CoV-2 virus was measured in broncho-alveolar lavage (BAL) and nasal swabs using an RT-PCR specific for subgenomic messenger RNA (sgmRNA), which are viral RNA intermediates believed to represent replicating virus^23–25^. The sham controls had a median peak of 3.74 log_10_ sgmRNA copies/mL in BAL (range: 2.5–4.03; Fig. 5a), which is consistent with a 4-5 log_10_ sgmRNA copies/mL peak observed in other studies conducted at the same facility with the same challenge dose and virus strain^26–28^. The vaccinated animals had a 2.04 log_10_ reduction in viral RNA in BAL. In particular, five out of six animals had viral loads below the quantitation limit of the assay (1.69 log_10_ sgmRNA copies/mL), one animal had a detectable low positive measure of 1.91 log_10_ sgmRNA copies/mL on one time point, day 4 post-challenge. In nasal swabs, vaccinated animals had a median peak viral load of 3.10 log_10_ sgmRNA copies/mL (range < 1.69–4.71; Fig. 5b). Varied viral loads measured for the two sham controls (6.41 and <1.69 log_10_ sgmRNA copies/mL) preclude a direct comparison to vaccinates. However, rhesus macaque sham controls challenged with the same dose and virus strain in the same facility had higher average peak viral loads (5.59–7.00 log_10_ sgmRNA copies/mL)^23,26–28^ in nasal swabs compared to the vaccinates in the present study. All vaccinated animals were SARS-CoV-2 antibody positive on the day of challenge (week 8); the anti-spike IgG titers increased the following challenge and was significantly higher 10 days post-challenge (median = 84775, range: 12669–148518) compared to 2 weeks after the third immunization (week 6; median = 10494, range: 5569–16525; *P* = 0.009) and on the day of challenge (week 8; median = 8314, range: 4598–11950; *P* = 0.002) (Fig. 5c).

### Breadth of neutralizing antibody responses

**Fig. 6:**
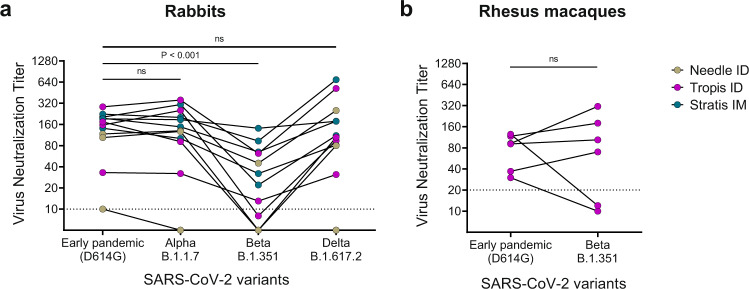
SARS-CoV-2 variant cross-neutralizing antibody responses induced by the pNTC-Spike vaccine after three immunizations (week 6). **a** pNTC-Spike vaccinated rabbit sera were evaluated for the Alpha variant (lineage B.1.1.7), Beta variant (lineage B.1.351), and Delta variant (lineage B.1.617.2) in a microneutralization assay at Statens Serum Institut, Denmark. **b** Sera from pNTC-Spike vaccinated rhesus macaques were evaluated in a plaque reduction neutralization test at BIOQUAL, Inc., USA. The variants tested were dependent on the viral isolates available at each institute. ns: not significant.

Cross-neutralizing antibody responses against SARS-CoV-2 variants of concern were determined for pNTC-Spike immunized rabbits and rhesus macaques after three immunizations (Fig. 6). The variants tested depending on the viral isolates available at the facilities where the animal studies were conducted. In rabbits, for all vaccine administration methods, pNTC-spike induced cross-reactive neutralizing antibodies against the Alpha (B.1.1.7), Beta (B.1.351), and Delta (B.1.617.2) variants in 91.7%, 66.7%, and 91.7% of vaccinated animals, respectively. With the optimized needle-free intradermal and intramuscular administrations, cross-neutralizing antibody responses increased to 100%, 77.7%, and 100%, respectively. Overall, the neutralization titers for the Alpha variant (median: 139, range: 5–136) and Delta variant (median: 108, range: 5–688) did not differ significantly from the early pandemic strain (median: 166, range: 10–275; *p* > 0.05 for both comparisons). However, the neutralization titers against the Beta variant (median: 27, range: 5–136) were reduced 6.1-fold (*p* = 0.0001). In rhesus macaques, pNTC-spike induced neutralizing antibodies against the Beta variant in 66.7% of animals; the neutralization titers for the early pandemic strain (median: 92, range: 30–95) and Beta variant (median: 87, range: 10–302) did not differ significantly (*p* = 0.563).

## Discussion

We developed a SARS-CoV-2-specific plasmid DNA vaccine candidate using platform optimization strategies to improve vaccine safety, antigen expression, potency, and immunogenicity. The candidate DNA vaccine is immunogenic in three different animal species, with a relatively small difference between smaller animals and nonhuman primates. The increased potency in nonhuman primates is likely attributed to a combination of improved vector design, optimization of antigen expression, and vaccine delivery through needle-free jet injection. Of note, the vaccine was equally immunogenic following administration, either intradermal or intramuscular, using needle-free jet delivery and were both superior to needle-and-syringe injection intradermal. The safety evaluation of pNTC-Spike in rabbits reiterated the known and established excellent safety profile of DNA vaccines in general.

We evaluated the protective effect of pNTC-Spike in a vaccination-challenge study of nonhuman primates. The macaque monkey is considered a suitable model for studying SARS-CoV-2 vaccine protection^23,29,30^. The animal species are susceptible to SARS-CoV-2 and develops virus-specific humoral and cellular immunity that may confer protection against subsequent infection. Naïve animals develop a systemic infection with high levels of SARS-CoV-2 replication in the upper and lower airways^23,30^. The disease course is generally mild and self-limiting, although pathological evidence of pneumonia is observed. Several, including now approved, SARS-CoV-2 vaccines have undergone evaluation in nonhuman primates. These include inactivated vaccines (PiCoVacc and BBIBP-CorV)^31,32^, viral vectored vaccines (ChAdOx1 and Ad26.COV2.S)^26,33^, mRNA vaccines (mRNA1273 and BNT162b2)^34^, DNA vaccines (INO-4800 and prototypes)^27,28^, and a subunit vaccine (NVX-CoV3273)^35^.

pNTC-Spike is one of three similar SARS-CoV-2 spike DNA vaccine candidates tested in rhesus macaques to date. The other includes a prototype vaccine expressing unmodified full-length wild-type spike (S) from within pcDNA3.1 + (a vector not approved for clinical use)^28^ and Inovio’s INO-4800 vaccine that comprises a clinical plasmid vector (pGX0001) encoding the full-length spike with an N-terminal IgE leader sequence and delivers intramuscularly by electroporation^27^. These vaccines were independently evaluated at the same nonhuman primate facility using the same challenge strain and challenge dose. The three DNA vaccines, administered intramuscular or intradermal, as two or three doses of 1–5 mg (without adjuvant) 2–4 weeks apart, all induced SARS-CoV-2 spike-specific immune responses in rhesus macaques and conferred protection against lower respiratory disease. Protection was not sterilizing, but likely the result of rapid control of viremia following challenge, as suggested by anamnestic antibody responses in protected prototype S and INO-4800 vaccinated animals^27,28^. In the present study, a 2 week interval between immunizations was deemed necessary to address the urgency for rapid vaccination during the pandemic; however, longer immunization intervals may likely increase immunogenicity and protection^36–38^. The previously reported SARS-CoV-2 plasmid DNA vaccines tested in nonhuman primates were well tolerated and none resulted in vaccine-associated enhanced respiratory disease (VAERD)^27,28^. Moreover, pNTC-Spike induced comparable antibody responses in small animals and nonhuman primates. These data support the known excellent safety profile of DNA vaccines and demonstrate that different SARS-CoV-2 spike DNA vaccines are immunogenic under various conditions without any adjuvants.

All SARS-CoV-2 vaccine formulations tested in nonhuman primates have conferred a substantial degree of protection to the immunized animals; however, sterilizing immunity was rarely achieved^26^. The common outcome, which extends to DNA vaccines, is a reduction of disease severity marked by lower viral loads, shorter duration of virus shedding, and reduced pathology in the lungs compared to control animals. The correlates of protection against infection and disease are currently unknown. Preliminary analyses suggest an important role for neutralizing antibodies (NAbs) in vaccine protection^26,28^. However, current macaque-challenge studies are insufficiently powered to conclude with confidence that NAbs levels at the time of challenge determine the outcome. Due to differences in neutralization assays used in the various vaccine studies, it is difficult to precisely compare NAb levels induced by the different vaccines; however, there is a trend towards other DNA vaccines inducing lower NAb titers compared to viral vectored vaccines, mRNA vaccines, and subunit vaccines^39^. Nevertheless, DNA vaccines were similarly efficacious to the majority of their counterparts in protecting vaccinated nonhuman primates against lower respiratory disease from SARS-CoV-2. This suggests either that low neutralizing antibody responses are sufficient or that other immune mechanisms such as cellular immunity or antibody Fc-mediated effector functions contribute to protection. Considering the presence of neutralizing antibodies following two immunizations with pNTC-Spike in rhesus macaques, we speculate that the DNA vaccine candidate may confer partial protection after two immunizations.

In the present study of vaccine-induced immunogenicity and protective effect, the nonhuman primate experiment was not designed specifically to assess safety, VAERD, or antibody-dependent enhancement of infection^40^. However, it is worth noting that the DNA vaccine elicited a Th1-biased rather than a Th2-biased T cell response in a Th1/Th2-balanced mouse (CB6F1) model alongside neutralizing antibodies; both features that associate with reduced histopathology in animal models^40–42^. Furthermore, pNTC-Spike vaccinated rhesus macaques did not demonstrate enhanced viral replication or clinical disease but complete or near-complete protection in BAL samples likely mediated by rapid immunological control of viral replication. To date, all approved and protective SARS-CoV-2 vaccines have a similar Th1-biased response^43^, and none of the human SARS-CoV-2 vaccines clinical trials testing nucleic acid vaccines, including mRNA and needle-free delivered plasmid DNA, has reported VAERD. On the contrary, these approved nucleic acid vaccines proved protective against disease^44–47^.

In addition to the homologous prime-boost regimen used, DNA vaccines can also complement other vaccines modalities in heterologous prime-boost strategies^5^. DNA is reportedly an excellent priming modality for both viral vector and protein subunit vaccines^48,49^. A DNA prime with a viral vector boost can circumvent or reduce the appearance of vector-specific neutralizing antibodies that may decrease viral vector vaccine efficacy. In the context of HIV vaccines, the modality combination has enhanced polyfunctional T cell responses in humans. Similarly, an HIV-specific DNA prime with a protein boost or co-administration of DNA and protein notably expanded the repertoire of functional antibody immune responses and enhanced CD4 + T cell responses compared to a protein prime—DNA boost regimen^5,50^.

Evaluating the improved DNA vaccine platform in clinical trials is warranted. It is well established this vaccine modality is safe in humans and demonstrated in independent studies that SARS-CoV-2 DNA vaccines are both immunogenic and protective in nonhuman primates. Clinical trials beyond phase I/II will be necessary to determine if this platform is a viable option in the emergency response to SARS-CoV-2, emerging SARS-CoV-2 variants of concern, or ‘Disease X’, the unknown future emergent pathogens that may potentially be equally or more devastating than COVID-19. Considerations to further optimize a vaccine regimen may include increasing the dose amount to reduce the number of doses required to induce sufficient quality immunity, although in affluent countries three immunizations may be feasible.

## Methods

### DNA vaccine

The study vaccine, pNTC-Spike, contains a DNA plasmid encoding an unmodified SARS-CoV-2 spike protein derived from the Wuhan-Hu-1 strain (MN908947). The human codon optimized SARS-CoV-2 spike sequence was synthesized by GeneArt (Thermo Fisher Scientific, Germany) and subcloned using *Eco*RI and *Xho*I into the NTC8685-eRNA41H vector backbone (Nature Technology Corporation, Lincoln, NE, USA). pNTC-Spike was produced by Nature Technology Corporation using an antibiotic-free selection procedure in NTC4862 *E. coli* cells (DH5α attλ::P5/6 6/6-RNA-IN-*Sac*V, Cm^r^)^12^ at 10 mg/mL in phosphate buffered saline (PBS). The plasmid preparation contained <2.0 EU/mg endotoxin, as determined by a Limulus Amoebocyte Lysate (LAL) test using the Endosafe nexgen-PTS LAL assay (Charles River, Wilmington, MA, USA). The construct was sequenced and tested for expression prior to use.

### Generation of antibodies specific for SARS-CoV-2 spike protein

Handling of laboratory animals for the production of monoclonal and polyclonal antibodies complied with the regulations of the German Animal Welfare Act and European legislation for the protection of animals used for scientific purposes (Directive 2010/63/EU). Immunizations of mice to generate monoclonal antibodies S1-1047 (IgG1) and S2-1254 (IgG1) received ethical approval by the State Office for Health and Social Affairs in Berlin (LAGeSo Berlin, Germany) under the registration number H129/19 (approval date 03/07/2019). NMRI mice (Charles River, Sulzfeld, Germany) were immunized three times with intervals of 3 weeks with 30 µg of recombinant SARS-CoV-2 spike domains S1 or S2, respectively (S1 = Cat. # REC31806, S2 = Cat. # REC31807, The Native Antigen Company, Oxford, UK) in Gerbu Adjuvans MM (GERBU Biotechnik GmbH, Heidelberg, Germany) according to the manufacturer´s instructions and finally boosted with 15 µg of the antigens in PBS at the last 3 days prior to fusion. Hybridoma cells were generated by the fusion of splenocytes from immunized mice with myeloma cells (P3-X63-Ag8.653, American Type Culture Collection)^51^. Cells were fused at 37 °C at a ratio of 4: 1 in polyethylene glycol 1500 (PEG, Roche Diagnostics, Mannheim, Germany) by slowly adding PEG (1 mL per 100 × 10^6^ splenocytes) to the pelleted cells, slow addition of RPMI 1640 (4 mL per 100 × 10^6^ splenocytes) and final addition of a larger volume of RPMI 1640 (10 mL per 100 × 10^6^ splenocytes). Cells were plated in a density of 20,000 splenocytes together with 20,000 BALB/c thymocytes as feeder cells in a volume of 200 µL per well of 96 well cell culture plates in RPMI 1640 media supplemented with 20% fetal calf serum, 50 µM 2-mercaptoethanol, 50 U/mL recombinant murine IL-6, 1% glutamine, 5.7 µM azaserine and 100 µM hypoxanthine. Starting at day 10 after fusion, antibodies from hybridoma supernatants underwent a stringent screening procedure employing e.g. ELISA and surface plasmon resonance spectroscopy to identify hybridoma clones with superior specificity, affinity, and broad applicability in different assays; selected clones were subcloned twice to ensure clonality. A rabbit polyclonal antibody (KSpike) was generated by subcutaneous immunization of a New Zealand rabbit with 25 µg of recombinant SARS-CoV-2 spike S1S2 protein (Cat. # 40589-V08B1, Sino biological, Bejing, China) for two times with an interval of 4 weeks. The IgG fractions were affinity purified from hybridoma culture supernatants or rabbit serum using Protein A or G columns, respectively, on an ÄKTA LC-instrument (ÄKTA, GE Healthcare Bio-Sciences AB, Uppsala, Sweden). The monoclonal antibodies S1-1047 and S2-1254 showed high specificity for their respective target domain in the spike protein of SARS-CoV-2 as shown by indirect ELISA using the rabbit pAb KSpike as control reagent (Supplementary Fig. 1).

### Western blot

The day before transfection, 1.2 × 10^5^ Vero E6 cells were seeded per well in a six well tissue culture plate with glass coverslips and incubated overnight at 37° C, 5% CO_2_. Vero E6 cells were transfected with 2 µg of pNTC-Spike using Fugene HD Transfection Reagent (Cat. # E2311, Promega, Madison, WI, USA). Cells were harvested 48 h post-transfection in lysis buffer (0.125 M NaCl, 20 mM Tris [pH 8.0], 0.5% Igepal). The protein content was determined by the Pierce™ BCA Protein Assay Kit (Cat. # 23227, Thermo Fisher, Waltham, MA, USA), and 25 µg cell lysate was separated on a Novex 10% Tris-Glycine Mini Gel (Cat. # XP00105BOX, Thermo Fisher) with the PageRuler™ Prestained Protein Ladder, 10 to 180 kDa (Cat. # 26616, Thermo Fisher). SARS-CoV-2 spike protein expression was detected with a 1:5000 dilution of anti-SARS-CoV-2 S1 and anti-SARS-CoV-2 S2 mouse monoclonal antibodies (S1-1047 and S2-1254, respectively) and visualized with a 1:5000 dilution of an HRP conjugated rabbit anti-mouse IgG polyclonal antibody (Cat. # P0161, Agilent, Santa Clara, CA, USA). A mouse monoclonal anti-β-actin (1:5000 dilution; Cat. # A1978, Sigma-Aldrich, Germany) was used to verify equal loading. As positive controls, a lysate preparation from Vero E6 cells infected with a SARS-CoV-2 clinical isolate and a commercial SARS-CoV-2 spike expressing plasmid (Cat. # VG40589-UT, Sino Biological, China) were used. The white light and chemoluminescence channel overlay of uncropped blots with molecular weight marker are presented in Supplementary Fig. 2.

### Immunofluorescence assay and microscopy

Vero E6 cells were seeded on coverslips in six-well plates and transfected with 2 µg of pNTC-Spike using Fugene HD Transfection Reagent (Cat. # E2311, Promega, Madison, WI, USA) or Polyfect Transfection reagent (Cat. # 301105, Qiagen GmbH, Hilden, Germany). Forty-eight hours post-transfection, cells were fixed at room temperature for 10 min in 4% paraformaldehyde (Cat. # HT501128-4L, Sigma-Aldrich), permeabilized at −20 °C for 10 min in 100% methanol. Cells were washed three times with Dulbecco’s phosphate buffered saline (DPBS) and blocked for 1 h at room temperature in blocking buffer (5% BSA in DPBS). The blocking buffer was aspirated, and cells were incubated for 2 h at 4 °C with a 1:1000 dilution of primary antibody in blocking buffer (S1-1047 or S2-1254). Cells were washed three times with DPBS and incubated for 1 h at room temperature in the dark with an Alexa Fluor 488 conjugated goat anti-mouse IgG (H + L) Trial Superclonal™ antibody (1:1000 dilution, Cat. # A28175, Thermo Fisher) in blocking buffer. Coverslips were washed three times with DPBS, dried on a paper towel, mounted with VECTASHIELD medium containing 4, 6-diamino-2-phenylindole hydrochloride (DAPI) (Cat. # H-1200-10, VECTOR Laboratories, Burlingame, CA, USA), and sealed with nail polish. Immunostained cells were observed with an Olympus BX61 fluorescence microscope, using the cellSens Entry software version 1.11 for image capture. Imaging software Image J, version 1.53j (NIH, USA) was used to merge images and visualize the results.

### Animals and study design

#### Mice

Eight week old female CB6F1 mice (Envigo, Netherlands), offspring of a cross between BALB/c and C57BL/6 mice, were randomly assigned to receive either 10 µg pNTC-Spike (*N* = 5), 50 µg pNTC-Spike (*N* = 5) or 50 µg of a vector control (*N* = 5). The unadjuvanted vaccine doses were prepared in PBS in a final volume of 50 µl and administered in two 25 µL injections per immunization. The mice were immunized via the intradermal route with needle injection at the base of the tail at weeks 0, 2, and 4.

#### Rabbits

Nine to ten week old female New Zealand white rabbits (Charles River, France) were immunized with 125 µg pNTC-Spike in PBS without adjuvant at weeks 0, 2, and 4. Three administration routes were evaluated in independent studies in the following order: intradermal route using the Tropis ID Needle-free Injection System (PharmaJet, Inc.) (*N* = 4); intradermal route using needle-and-syringe (*N* = 3); intramuscular route in a single dose of 500 µL using the Stratis Needle-free Injection System (PharmaJet, Inc.) (*N* = 5). In the latter study, vaccine safety was observed.

#### Nonhuman primates

Eight male and female adult rhesus macaques (*Macaca mulatta*), 2–8 years old (mean: 4 years), were randomly divided into two groups: pNTC-Spike vaccinates (*N* = 6) and sham controls (*N* = 2). Animals received three immunizations of 2 mg DNA each at weeks 0, 2, and 4. The unadjuvanted vaccine was administered via the intradermal route using the Tropis ID needle-free injection system (PharmaJet, Inc.) with four 100 μL doses per immunization, equally distributed over the left and right scapula region. The interval between last immunization to viral challenge varies for the different SARS-CoV-2 vaccine candidates (adenoviral vector, mRNA, live attenuated, protein and DNA) evaluated in nonhuman primates (median: 25 days; range: 14–77 days)^39^. To enable comparison with the latter, we selected an interval of 28 days between the last immunization and challenge. At week 8, all eight animals were challenged with 1.0 × 10^5^ TCID_50_ (1.2 × 10^8^ RNA copies, 1.1 × 10^4^ PFU) SARS-CoV-2 (strain nCoV-WAI-2020; MN985325.1; BEI Resources, Manassas, VA, USA). In similar studies conducted at the same facility, BIOQUAL, Inc., the 1.0 × 10^5^ TCID_50_ challenge dose consistently resulted in infection of SARS-CoV-2 immune naïve rhesus macaques (*N* = 28, total) with detectable viral loads by 2 days post-challenge in BAL and nasal swabs.^23,26–28^ The challenge stock was propagated at BIOQUAL, Inc. in Vero E6 cells from a seed stock obtained by Kenneth Plante, World Reference Center for Emerging Viruses and Arboviruses, UTMB, Galveston, TX (lot no. TVP 23156). The stock was deep sequenced (SRA accession no. SRR12749718) by Shelby O’Connor’s laboratory, Univ. Wisconsin-Madison, WI. Sequencing confirmed the expected sequence identity. The virus was diluted in PBS and administered as 1 mL by the intranasal (IN) route and 1 mL by the intratracheal (IT) route.

The mice and rabbits were housed in a pathogen-free and climate-controlled animal facility at Statens Serum Institut, Denmark. All cages were provided with bedding material and environmental enrichment. Animals had access to water and a standard pelleted diet *ad libitum*. Animal husbandry and procedures comply with the Danish legislation, which is based on the EU Directive 2010/63/EU on the protection of animals used for scientific purposes. The experiments received ethical approval by The Animal Experimentation Council, the National Competent Authority within this field (approval number 2017-15-0201-01322), and were supervised by the laboratory animal veterinarians at Statens Serum Institut. The nonhuman primates were housed at BIOQUAL Inc. (Rockville, MD) and associated animal studies conducted in compliance with relevant local, state, and federal regulations and received ethical approval by the Institutional Animal Care and Use Committee (IACUC). The assays used to quantitate vaccine-induced immune responses in the small animals and rhesus macaques were performed at Statens Serum Institut and BIOQUAL, Inc., respectively, and were depended on the assays available at each institute.

### Mouse and rabbit antibody enzyme-linked immunosorbent assay (ELISA)

Spike- and RBD-specific binding immunoglobulin G (IgG) titers were determined by standard ELISA. In brief, Nunc™ MaxiSorp™ plates were coated with 100 µL of 4 µg/mL recombinant SARS-CoV-2 spike S1S2 protein (Cat. # 40589-V08B1; Sino biological) or 4 µg/mL SARS-CoV-2 spike RBD protein (Cat. # 40592-V08B; Sino biological) at 4 °C overnight. In consecutive order, with wash steps in between, plates were incubated at room temperature on an orbital shaker with 150 µL of blocking buffer (SSI Dilution Buffer [Cat. # 1322, SSI Diagnostica], 2% skim milk), 100 µL of 5-fold serial dilutions of a mouse or rabbit sera (1:20 to 1:1562500), 100 µL of horseradish peroxidase (HRP) conjugated goat anti-mouse IgG antibody (1:10000 dilution) or mouse-anti-rabbit IgG antibody (1:2000 dilution) (Cat. # A4416 and A1949, respectively; Sigma-Aldrich), and 3,3′, 5,5′-Tetramethylbenzidine (TMB) One Substrate (Cat. # 4380, KemEnTec, Denmark). TMB reaction was stopped with H_2_SO_4_, and absorbance read at 450 nm using 620 nm as a reference on a FLUOstar Microplate Reader (BMG LABTECH, Germany). Each wash step comprised three washes with 250 µL wash buffer (PBS with 0.05% Tween20) for 1 min.

### Spike ectodomain-specific antibody ELISA

White Nunc™ MaxiSorp™ microtiter plates were coated with 100 µL of 1 µg/mL recombinant SARS-CoV-2 Spike His-tag protein (Cat. # 10549-CV-100; R&D Systems) at 4 °C overnight. In consecutive order, with wash steps in between, plates were incubated with 150 µL of blocking buffer (Dilution Buffer pH 7.2 [Cat. # 1322, SSI Diagnostica], 2% bovine serum albumin, and 0.1% Tween20), 100 µL of 5-fold serial dilutions of rabbit sera (1:20 to 1:1562500), 100 µL of HRP conjugated mouse-anti-rabbit IgG antibody (1:2000 dilution) (Cat. # A1949 Sigma-Aldrich), and BM Chemiluminescent Substrate (Cat. # 11582950001, Sigma-Aldrich). Luminescence was read on a FLUOstar Microplate Reader (BMG LABTECH, Germany). Each wash step comprised three washes with 250 µL wash buffer (PBS with 0.05% Tween20) for 30 s. ELISA end-point titers were calculated from a four-parameter logistic regression curve in GraphPad Prism 8.3.0, using the reciprocal serum dilution that yielded an absorbance above a positive cut-off value calculated for each group of animals based on absorbances measured on day 0 at a serum dilution of 1:20 that is (mean absorbance for all animals) + (3 × standard deviation of the absorbance measured for all animals).

### Virus microneutralization test

A 2-fold serial dilution of heat-inactivated serum/plasma samples were mixed with 300 × TCID_50_ SARS-CoV-2 virus, as determined from a virus titration 96 h post-inoculation. The solution was incubated for 1 h at 37 °C, 5% CO_2_, and added to Vero E6 cells (kindly provided by Bjoern Meyer, Institut Pasteur, Paris, France) in a 96 well tissue culture plate seeded with 10^4^ cells per well the day prior. The inoculated cells were incubated at 37 °C, 5% CO_2_ for 24 h, and the inhibition of virus infection in the presence of serum measured in a standard ELISA targeting the SARS-CoV-2 nucleocapsid protein. The culture medium was removed from the infected Vero E6 cell monolayers and the cells washed twice with 100 μL PBS. The cells were fixed with cold 80% (v/v) acetone in PBS for 10 min. Following three wash steps with wash buffer (PBS containing 1% (v/v) Triton-X100) for 30 s, a 100 μL of a SARS-CoV-2 nucleocapsid protein monoclonal antibody was added and incubated for 5 min on an orbital shaker (300 rpm) at room temperature and subsequently for 1 h at 37°C. For testing rabbit sera, the mouse monoclonal antibody clone 7E1B was used (1:4000 dilution; Cat. # BSM-41414M, Bioss, Woburn, Massachusetts, USA); for testing mouse sera, a rabbit monoclonal antibody was used (1:2500; Cat. # 40143-R019, Sino Biological, China). The plates were washed and incubated with 100 μL of either a 1:6000 diluted mouse-anti-rabbit IgG HRP conjugate antibody (Cat. # A1949, Sigma-Aldrich) or a 1:10000 diluted goat anti-mouse IgG (H + L) cross-adsorbed HRP conjugate antibody (Cat. # A16078; Invitrogen, Waltham, Massachusetts, USA) for 5 min on an orbital shaker (300 rpm) at room temperature and subsequently for 1 h at 37 °C. The plates were washed five times with wash buffer for 30 s, followed by three washes with deionized water. A 100 μL TMB One Substrate (Cat. # 4380, KemEnTec, Denmark) was added and incubated for 15 min. The reaction was stopped with H_2_SO_4_, and absorbance read at 450 nm using 620 nm as a reference on a FLUOstar Microplate Reader (BMG LABTECH, Germany).

Included on each microneutralization plate were quadruplicate wells containing cells with 300 × TCID_50_ SARS-CoV-2 virus without serum (virus control) and quadruplicate wells containing cells with virus diluent only (cell control). The neutralization antibody titer was determined for each serum sample as the interpolation of a four-parameter logistic regression curve with the 50% virus level cut-off calculated for each assay plate: [(mean OD of virus control wells) + (mean OD of cell control wells)]/2. The reciprocal serum dilution corresponding to that well is reported as the 50% neutralization antibody titer for that sample. The microneutralization assay is validated and has comparable performance to other live virus neutralization assays established at different European laboratories (laboratory 4 in ref. ^52^). Virus neutralization was measured for the following SARS-CoV-2 viruses: early pandemic (lineage B.1) strain SARS-CoV-2/Hu/Denmark/SSI-H1; Alpha variant (lineage B.1.1.7) strain SARS-CoV-2/Hu/Denmark/SSI-H14; Beta variant (lineage B.1.351) strain hCoV-19/Netherlands/NoordHolland_10159/2021 (Cat. # 014V-04058, European Virus Archive—Global, Marseille, France); and the Delta variant (lineage B.1.617.2) strain SARS-CoV-2/Hu/Denmark/SSI-H11. Strains from Denmark were isolated at Statens Serum Institut from clinical samples on Vero E6 cells. All virus stocks were deep sequenced to confirm identity, confirm the absence of cell culture-derived mutations, and the presence of lineage-specific mutations in the spike protein. The comparison between strains was done on the same day using a single dilution for each serum sample.

### Mouse and rabbit splenocyte isolation, restimulation, and cytokine quantification

Following excision, the spleens were submerged in RPMI medium and processed aseptically within an hour. The spleens were homogenized through a 70 µm cell strainer using a syringe plunger. The single-cell suspensions were washed twice with cold PBS followed by lysis of red blood cells using Red Blood Cell Lysis buffer (Cat. # R7757, Sigma-Aldrich). The splenocytes were resuspended in culture medium (RPMI containing 10 % fetal bovine serum, 1 % Penicillin and Streptomycin, 1 mM sodium pyruvate, 10 mM HEPES and 50 µM 2-Mercaptoethanol). A total of 8 × 10^5^ splenocytes were stimulated in duplicate with 2 µg/mL SARS-CoV-2 spike S1S2 protein (Cat. # 40589-V08B1; Sino biological), 2 µg/mL SARS-CoV-2 spike RBD protein (Cat. # 40592-V08B; Sino biological), 5 µg/mL concanavalin A (Cat. # C0412, Sigma-Aldrich) as a positive control, or culture media as mock stimulated control. The re-stimulated splenocytes were kept under standard tissue culture conditions (37˚C with 5% CO_2_) for 48 h. The level of secreted interferon-γ (IFN-γ), interleukin 5 (IL-5), or interleukin 17 (IL-17) in clarified cell culture supernatants were determined by cytokine ELISA (Cat. # 3321-1H-6, 3391-1H-6, 3521-1H-6, 3110-1H-6; MABTECH AB, Sweden) according to the manufacturer’s instructions. For the rabbit IFN-γ ELISpot (Cat. # 3110-4HPW-10; MABTECH AB, Sweden), a total of 1 × 106 splenocytes were stimulated for 18 h using the aforementioned antigen stimulations and tissue culture conditions. The secretion of IFN-γ was determined according to the manufacturer’s instructions and spots were counted using CTL Immunospot® analyzer and ImmunSpot® Software (version 7.0.22.1).

### Nonhuman primate antibody enzyme-linked immunosorbent assay (ELISA)

SARS-CoV-2 spike protein-specific IgG in serum was quantified in duplicate by ELISA. In brief, Nunc™ MaxiSorp™ microtiter plates were coated with 50 µL of 2 μg/mL recombinant SARS-CoV-2 spike S1S2 protein (Cat. # 40589-V08B1, Sino Biological) in PBS and incubated overnight at 4°C. Plates were washed five times with wash buffer (0.05% Tween20 in PBS) and blocked with 100 μL 1% bovine serum albumin in PBS for 2 h at room temperature. The block solution was discarded and 100 μL of serial 4-fold dilutions of serum starting at a 1:20 dilution were added to the wells, followed by a 1 h incubation at room temperature. Plates were washed three times with wash buffer and incubated for 1 h at room temperature with 50 µL a 1:10000 dilution of goat anti-monkey IgG (H + L) (Cat. # PA1-84631, Invitrogen). Plates were washed five times with wash buffer and once with PBS, followed by the addition of 100 μL of SureBlue TMB 1-Component Microwell Peroxidase Substrate (Cat. # 5120-0075, SeraCare, Milford, MA, USA). The reaction was stopped after 10 min with the addition of 100 μL TMB Stop solution per well. The absorbance was measured at 450 nm using 620 nm as a reference. ELISA end-point titers were defined as the highest reciprocal serum dilution that yielded an absorbance >0.300.

### Plaque reduction neutralization test (PRNT)

The PRNT was performed in six-well tissue culture plates seeded with 1.75 × 10^5^ Vero E6 cells (Cat. # CRL-1586, ATCC, Manassas, VA, USA) per well the day before. Serum samples were heat-inactivated at 56 °C for 30 min and tested in duplicate in a three-fold serial dilution ranging from 1:20 to 1:4860. Each serum dilution was pre-incubated with 30 PFU SARS-CoV-2 for 1 h at 37 °C before addition to the Vero76 monolayers. After an incubation of 1 h at 37 °C, the supernatants containing the serum/virus mixture were removed and the monolayer washed once with PBS before overlaying with a semi-solid culture medium. Following a 3 day incubation at 37 °C with 5% CO_2_, the cells were fixed and stained with crystal violet. The reciprocal of the serum dilutions causing plaque reductions of 90% (PRNT_90_) and 50% (PRNT_50_) were recorded as titers. Virus neutralization was measured for the early pandemic SARS-CoV-2 strain nCoV-WAI-2020 (Cat. # NR-52281, BEI Resources, Manassas, VA, USA) and the Beta variant (lineage B.1.351) strain 2019-nCoV/South Africa/KRISP-K005325/2020 (Cat. # NR-54009, BEI Resources). All virus stocks were deep sequenced to confirm identity.

### Subgenomic SARS-CoV-2 RNA assay

Replicating the SARS-CoV-2 virus was detected and measured using a real-time RT-PCR assay targeting viral replication intermediates not packaged into virions^24^. RNA was extracted using the QIAamp Viral RNA Mini Kit (Cat. # 52904, Qiagen, Hilden, Germany). The SARS-CoV-2 E gene subgenomic messenger RNA (sgmRNA) was detected using a leader-specific primer (SG-F: 5′-CGATCTTGTAGATCTGTTCCTCAAACGAAC-3′) located upstream of SARS-CoV-2 ORF1a and a reverse primer (SG-R: 5′-ATATTGCAGCAGTACGCACACACA-3′) and probe (FAM-5′-ACACTAGCCATCCTTACTGCGCTTCG-3′-BHQ) specific to the E gene. PCR amplification was performed using the SensiFAST™ Probe Lo-ROX One-Step Kit (Cat. # BIO-78005, Meridian Bioscience, Cincinnati, OH, USA) on an Applied Biosystems 7500 Real-Time PCR instrument with the following program: 48 °C for 30 min, 95 °C for 10 min followed by 40 cycles of 95 °C for 15 s, and 1 min at 55 °C. Subgenomic RNA copies were calculated from a standard curve representing serially diluted plasmid DNA containing the target sequence.

### Statistical analyses

Each measurement represents a single animal. Antibody positive and negative responses were determined using a 99.9% confidence interval cut-off value calculated for each serum dilution from the serum controls included in the assays^53^. Variation in paired continuous variables between multiple time points were compared using the non-parametric Friedman test with Dunn’s correction for multiple comparisons. For the latter, adjusted *p*-values are reported. The Wilcoxon rank sum test was performed where data for only two time points were available. All statistical tests were two-tailed. Statistical analyses and graphing were done with GraphPad PRISM version 8.3.0. (GraphPad Software Inc., San Diego, CA).

### Reporting summary

Further information on research design is available in the Nature Research Reporting Summary linked to this article.

## Supplementary information

Supplementary Information

Reporting Summary

## Data Availability

Data are available from the corresponding author upon request.
