# Supplementary material for: Preclinical evaluation of a candidate naked plasmid DNA vaccine against SARS-CoV-2
Source: NPJ Vaccines. 2021 Dec 20;6:156. doi: 10.1038/s41541-021-00419-z (PMC8688418; doi:10.1038/s41541-021-00419-z)
Supplement: Supplementary file 1 — Supplementary Information [file 41541_2021_419_MOESM1_ESM.pdf]

Supplementary Information

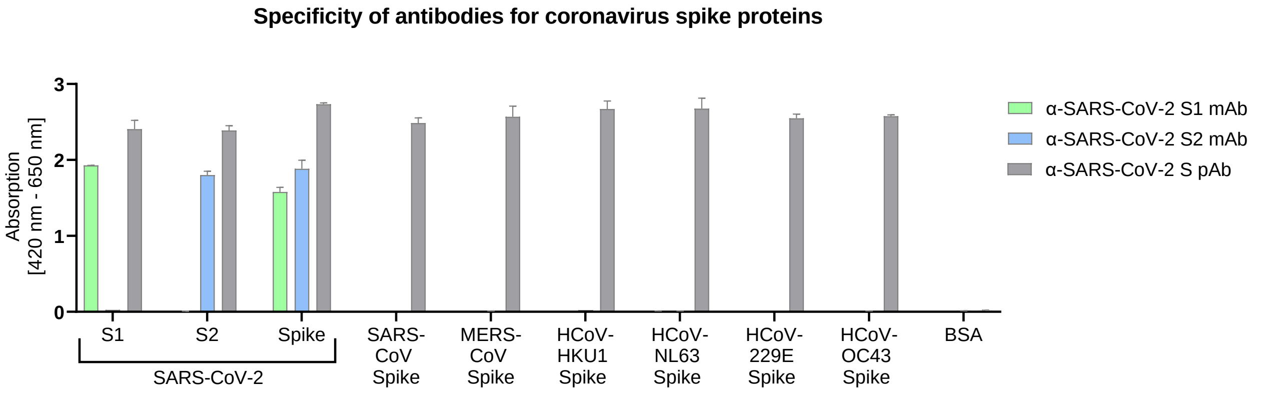

Supplementary Fig. 1. Specificity of monoclonal antibodies S1-1047 and S2-1254 in ELISA. Nunc™ MaxiSorp™ plates were coated with 50 µL of 500 ng/mL of the indicated recombinant spike proteins in PBS containing 1 µg/mL BSA overnight at 4 °C. The following steps were performed at room temperature with precedent fourfold washing steps with PBS supplemented with 0.1% Tween 20 (PBS-T). Wells were blocked with 2% skimmed milk powder (Merck, Darmstadt, Germany) in PBS-T for 1 h, incubated with 10 µg/mL of mouse monoclonal antibodies S1-1047 (α-SARS-CoV2 S1), S2-1254 (α-SARS-CoV2 S1), or rabbit polyclonal antibody KSpike (α-SARS-CoV2 S), all generated in this work, for 1 h, followed by HRP-labeled goat-anti-mouse IgG (Fcγ)- or goat anti-rabbit IgG (H+L)-specific antibodies (1:2500; Dianova, Hamburg, Germany) for 30 min. After a final eightfold washing step, plates were incubated with 100 µL per well of 3,3′,5,5′-tetramethylbenzidine (TMB, SeramunBlau slow; Seramun, Heidesee, Germany) and the reaction was stopped by adding 100 µL of 0.25 M H_2SO_4 per well. Absorbance at 420 nm referenced to 620 nm was measured by an ELISA reader (Tecan; Crailsheim, Germany). Results are given as mean ± standard deviation of two replicates. S1 = Cat. # REC31806, S2 = Cat. # REC31807 (both The Native Antigen Company), Spike = Cat. # 40589-V08B1 (Sino biological). SARS-CoV Spike = Cat. # SPN-S52H5 (Acro Biosystems), MERS-CoV Spike = Cat. # 40069-V08B, HCoV-HKU1 Spike = Cat. # 40606-V08B, HCoV-NL63 Spike = Cat. # 40604-V08B, HCoV-229E Spike = Cat. # 40605-V08B, HCoV-OC43 Spike = Cat. # 40607-V08B (all Sino biological). The monoclonal antibodies were applied to verify protein expression from the DNA vaccine candidate (Fig. 1b).

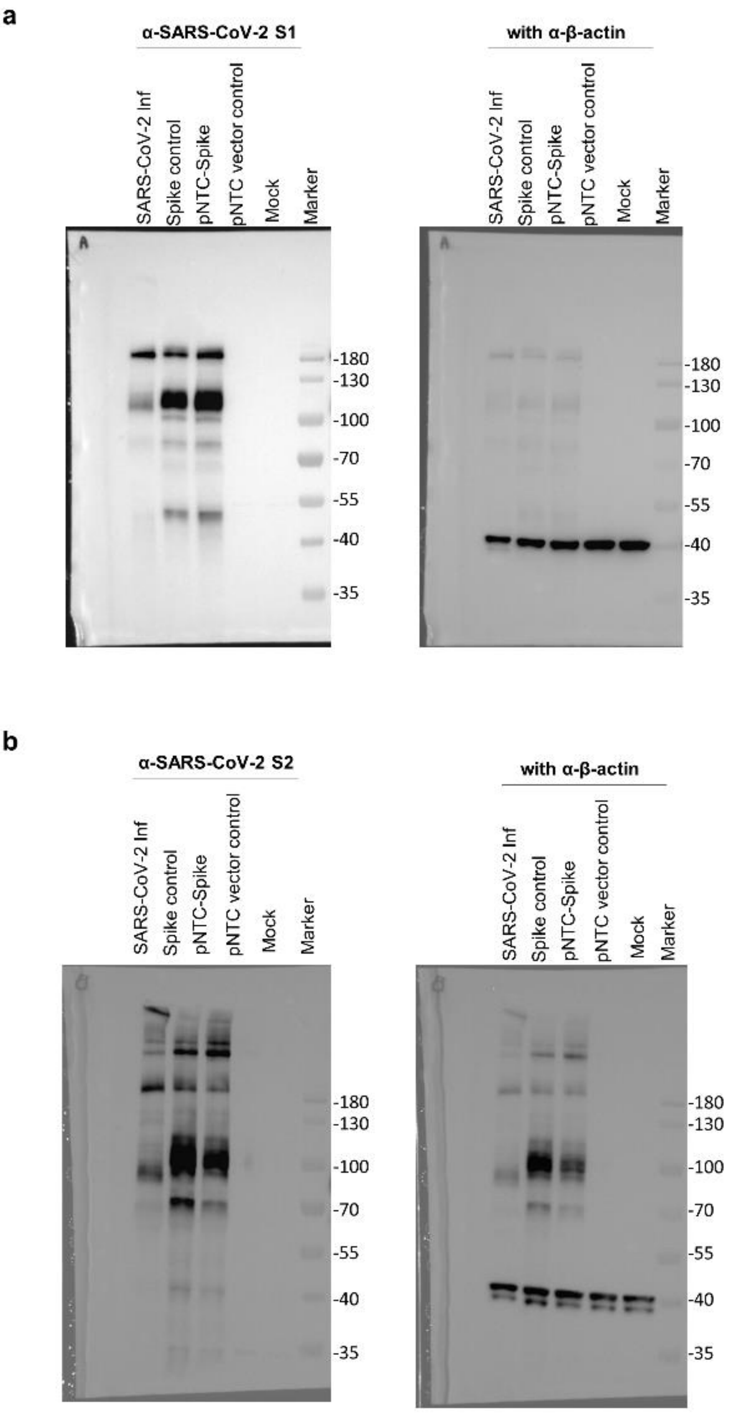

Supplementary Fig. 2. Raw Western Blot images with molecular marker. Protein expression from the DNA vaccine candidate was confirmed by DNA lipofection into Vero E6 cells followed by western blotting using anti-S1 (a) and anti-S2 (b) detecting mouse monoclonal antibodies S1-1047 and S2-1254, respectively. Lanes: 1) SARS-CoV-2 infected Vero E6 extract 2) Spike expressing plasmid (commercial positive control), 3) pNTC-Spike, 4) NTC vector control, and 5) VERO E6 (negative control) and 6) molecular marker. Blots were washed and subsequently re-probed with a β-actin antibody (right panels) to detect 42 kDa β-actin expression as loading control.

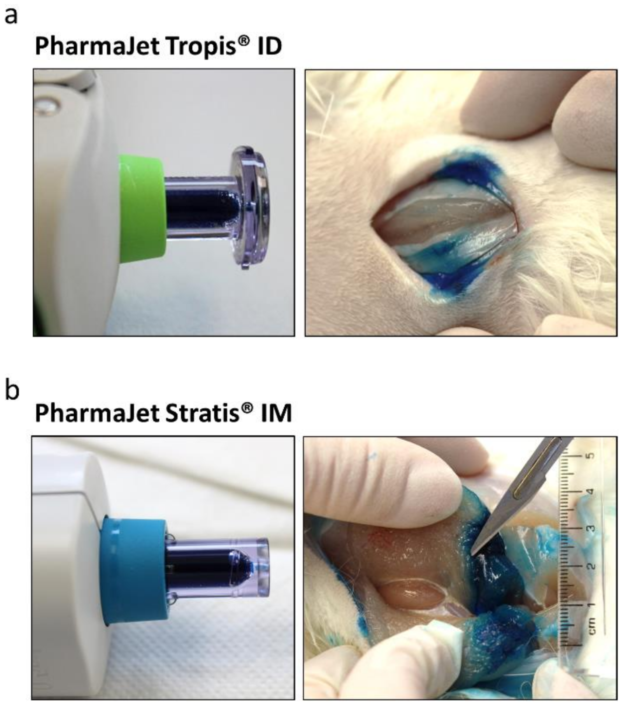

Supplementary Fig. 3. Intradermal and intramuscular needle-free vaccination of rabbits with the PharmaJet Tropis® ID and Stratis® IM, respectively. The needle-free devices are calibrated for human use. To confirm that these devices deliver liquid to the correct compartment in rabbits, we tested each device on a New Zealand white rabbit using the vaccine diluent, phosphate buffered saline (PBS), with a blue dye to visualize penetrance. The volume administered is standard at 100 µL for the Tropis ID and 500 µL for the Tropis ID. The site of administration was shaved. a) The Tropis ID device delivered PBS into rabbit skin without penetrating the underlying muscle. b) The Stratis IM device delivered 500 µL of PBS through the skin, penetrating approximately 2 cm deep into the thigh muscle.
