# Supplementary material for: Preclinical evaluation of a candidate naked plasmid DNA vaccine against SARS-CoV-2
Source: NPJ Vaccines. 2021 Dec 20;6:156. doi: 10.1038/s41541-021-00419-z (PMC8688418; doi:10.1038/s41541-021-00419-z)
Supplement: Supplementary file 2 — Reporting Summary [file 41541_2021_419_MOESM2_ESM.pdf]

nature portfolio

Corresponding author(s): *Prof Anders Fomsgaard*

Last updated by author(s): *Aug 11, 2021*

Reporting Summary

Nature Portfolio wishes to improve the reproducibility of the work that we publish. This form provides structure for consistency and transparency in reporting. For further information on Nature Portfolio policies, see our Editorial Policies and the Editorial Policy Checklist.

Statistics

For all statistical analyses, confirm that the following items are present in the figure legend, table legend, main text, or Methods section.

n/a	Confirmed	
☐	☒	The exact sample size (*n*) for each experimental group/condition, given as a discrete number and unit of measurement
☐	☒	A statement on whether measurements were taken from distinct samples or whether the same sample was measured repeatedly
☐	☒	The statistical test(s) used AND whether they are one- or two-sided<br>*Only common tests should be described solely by name; describe more complex techniques in the Methods section.*
☒	☐	A description of all covariates tested
☐	☒	A description of any assumptions or corrections, such as tests of normality and adjustment for multiple comparisons
☐	☒	A full description of the statistical parameters including central tendency (e.g. means) or other basic estimates (e.g. regression coefficient) AND variation (e.g. standard deviation) or associated estimates of uncertainty (e.g. confidence intervals)
☐	☒	For null hypothesis testing, the test statistic (e.g. *F*, *t*, *r*) with confidence intervals, effect sizes, degrees of freedom and *P* value noted<br>*Give P values as exact values whenever suitable.*
☒	☐	For Bayesian analysis, information on the choice of priors and Markov chain Monte Carlo settings
☒	☐	For hierarchical and complex designs, identification of the appropriate level for tests and full reporting of outcomes
☒	☐	Estimates of effect sizes (e.g. Cohen's *d*, Pearson's *r*), indicating how they were calculated

Our web collection on statistics for biologists contains articles on many of the points above.

Software and code

Policy information about availability of computer code

Data collection: *No software used for data collection*

Data analysis: *Fluorescence microscopy: cellSens Entry software version 1.11 for image capture; imaging software Image J, version 1.53j (NIH, USA) to merge images and visualize results.*
CTL ImmunSpot® Software (version 7.0.22.1).
Statistical analyses and graphing were done with GraphPad PRISM version 8.3.0. (GraphPad Software Inc., San Diego, CA).

For manuscripts utilizing custom algorithms or software that are central to the research but not yet described in published literature, software must be made available to editors and reviewers. We strongly encourage code deposition in a community repository (e.g. GitHub). See the Nature Portfolio guidelines for submitting code & software for further information.

Data

Policy information about availability of data

All manuscripts must include a data availability statement. This statement should provide the following information, where applicable:

- Accession codes, unique identifiers, or web links for publicly available datasets
- A description of any restrictions on data availability
- For clinical datasets or third party data, please ensure that the statement adheres to our policy

Data are available from the corresponding author upon request.

Field-specific reporting

Please select the one below that is the best fit for your research. If you are not sure, read the appropriate sections before making your selection.

☒ Life sciences ☐ Behavioural & social sciences ☐ Ecological, evolutionary & environmental sciences

For a reference copy of the document with all sections, see nature.com/documents/nr-reporting-summary-flat.pdf

Life sciences study design

All studies must disclose on these points even when the disclosure is negative.

Sample size	*Mice and rabbits - Sample size was determined based on previous experience measuring the immunogenicity of vaccines where the numbers used were sufficient for statistical analyses.<br>NHP - The sample size for vaccinated animals was based on experience with SARS-CoV-2 vaccines in the model. The sample size for controls were determined by cost and the availability of historical data for more than 20 control animals challenged with the same viral dose and strain in the same facility.*
Data exclusions	*No data were excluded*
Replication	*Serological analysis: NHP serum samples were analyzed in duplicate from each animal for each timepoint<br>Mouse neutralizing antibody responses: serum samples were analyzed in duplicate<br>ELISpot assays were performed in duplicate<br>All repeats were successful.*
Randomization	*Animals were randomly assigned to groups*
Blinding	*Blinding was done for the following personnel:<br>- Person scoring animals daily<br>- Technicians performing immunological assays*

Behavioural & social sciences study design

All studies must disclose on these points even when the disclosure is negative.

Study description	*Not applicable*
Research sample	*Not applicable*
Sampling strategy	*Not applicable*
Data collection	*Not applicable*
Timing	*Not applicable*
Data exclusions	*Not applicable*
Non-participation	*Not applicable*
Randomization	*Not applicable*

Ecological, evolutionary & environmental sciences study design

All studies must disclose on these points even when the disclosure is negative.

Study description	*Not applicable*
Research sample	*Not applicable*
Sampling strategy	*Not applicable*
Data collection	*Not applicable*
Timing and spatial scale	*Not applicable*

Data exclusions	*Not applicable*
Reproducibility	*Not applicable*
Randomization	*Not applicable*
Blinding	*Not applicable*

Did the study involve field work? ☐ Yes ☒ No

Field work, collection and transport

Field conditions	*Not applicable*
Location	*Not applicable*
Access & import/export	*Not applicable*
Disturbance	*Not applicable*

Reporting for specific materials, systems and methods

We require information from authors about some types of materials, experimental systems and methods used in many studies. Here, indicate whether each material, system or method listed is relevant to your study. If you are not sure if a list item applies to your research, read the appropriate section before selecting a response.

Materials & experimental systems

n/a	Involved in the study	
☐	☒	Antibodies
☐	☒	Eukaryotic cell lines
☒	☐	Palaeontology and archaeology
☐	☒	Animals and other organisms
☒	☐	Human research participants
☒	☐	Clinical data
☒	☐	Dual use research of concern

Methods

n/a	Involved in the study	
☒	☐	ChIP-seq
☒	☐	Flow cytometry
☒	☐	MRI-based neuroimaging

Antibodies

Antibodies used	*Mouse monoclonal antibodies S1 1047 (IgG1) and S2 1254 (IgG1), Robert Koch Institute*<br>*HRP conjugated rabbit anti-mouse IgG polyclonal antibody (Cat. # P0161, Agilent, Santa Clara, CA, USA)*<br>*Mouse monoclonal anti-β-actin (Cat # A1978, Sigma-Aldrich)*<br>*Alexa Fluor 488 conjugated goat anti-mouse IgG (H+L) Trial Superclonal™ antibody (Cat. # A28175, ThermoFisher)*<br>*HRP conjugated goat-anti-mouse IgG antibody (Cat. # A4416, Sigma-Aldrich, Germany)*<br>*HRP conjugated mouse-anti-rabbit IgG antibody (Cat. # A1949, Sigma-Aldrich, Germany)*<br>*Goat anti-monkey IgG (H+L) (Cat. # PA1-84631, Invitrogen)*
Validation	*The monoclonal antibodies S1-1047 and S2-1254 were validated in-house, showing a high specificity for their respective target domain in the spike protein of SARS-CoV-2 as shown by indirect ELISA using the rabbit pAb KSpike as control reagent (Supplementary Fig. S1).*<br>*All other antibodies were validated by the supplier.*

Eukaryotic cell lines

Policy information about cell lines

Cell line source(s)	*Vero E6 cells, obtained from Pasteur Institute, France in April 2020*
Authentication	*Not authenticated in-house.*
Mycoplasma contamination	*Mycoplasma testing confirmed negative at regular intervals.*
Commonly misidentified lines (See ICLAC register)	*No commonly misidentified cell lines were used.*

Palaeontology and Archaeology

Specimen provenance	*Not applicable*
Specimen deposition	*Not applicable*
Dating methods	*Not applicable*

☐ Tick this box to confirm that the raw and calibrated dates are available in the paper or in Supplementary Information.

Ethics oversight	*Not applicable*

Note that full information on the approval of the study protocol must also be provided in the manuscript.

Animals and other organisms

Policy information about studies involving animals; ARRIVE guidelines recommended for reporting animal research

Laboratory animals	*Mice - Eight week old female CB6F1 mice (Envigo, Netherlands), offspring of a cross between BALB/c and C57BL/6 mice<br>Rabbits - Nine to ten week old female New Zealand White rabbits (Charles River, France)<br>NHP - Eight male and female adult rhesus macaques (Macaca mulatta), 2 to 8 years old (mean: 4 years)*
Wild animals	*No wild animals were used.*
Field-collected samples	*No field collected samples were used.*
Ethics oversight	*Mice and rabbits - Animal husbandry and procedures were carried out in accordance with the Animal Experimentation Act of Denmark and the European Convention ETS 123.<br>NHP - Animal studies conducted in compliance with relevant local, state, and federal regulations and were approved by the Institutional Animal Care and Use Committee (IACUC).*

Note that full information on the approval of the study protocol must also be provided in the manuscript.

Human research participants

Policy information about studies involving human research participants

Population characteristics	*Not applicable*
Recruitment	*Not applicable*
Ethics oversight	*Not applicable*

Note that full information on the approval of the study protocol must also be provided in the manuscript.

Clinical data

Policy information about clinical studies

All manuscripts should comply with the ICMJE guidelines for publication of clinical research and a completed CONSORT checklist must be included with all submissions.

Clinical trial registration	*Not applicable*
Study protocol	*Not applicable*
Data collection	*Not applicable*
Outcomes	*Not applicable*

Dual use research of concern

Policy information about dual use research of concern

Hazards

Could the accidental, deliberate or reckless misuse of agents or technologies generated in the work, or the application of information presented in the manuscript, pose a threat to:

No	Yes	
☒	☐	Public health
☒	☐	National security
☒	☐	Crops and/or livestock
☒	☐	Ecosystems
☒	☐	Any other significant area

Experiments of concern

Does the work involve any of these experiments of concern:

No	Yes	
☒	☐	Demonstrate how to render a vaccine ineffective
☒	☐	Confer resistance to therapeutically useful antibiotics or antiviral agents
☒	☐	Enhance the virulence of a pathogen or render a nonpathogen virulent
☒	☐	Increase transmissibility of a pathogen
☒	☐	Alter the host range of a pathogen
☒	☐	Enable evasion of diagnostic/detection modalities
☒	☐	Enable the weaponization of a biological agent or toxin
☒	☐	Any other potentially harmful combination of experiments and agents

ChIP-seq

Data deposition

☐ Confirm that both raw and final processed data have been deposited in a public database such as GEO.

☐ Confirm that you have deposited or provided access to graph files (e.g. BED files) for the called peaks.

Data access links<br>*May remain private before publication.*	*Not applicable*
Files in database submission	*Not applicable*
Genome browser session<br>(e.g. UCSC)	*Not applicable*

Methodology

Replicates	*Not applicable*
Sequencing depth	*Not applicable*
Antibodies	*Not applicable*
Peak calling parameters	*Not applicable*
Data quality	*Not applicable*
Software	*Not applicable*

Flow Cytometry

Plots

Confirm that:

☐ The axis labels state the marker and fluorochrome used (e.g. CD4-FITC).

☐ The axis scales are clearly visible. Include numbers along axes only for bottom left plot of group (a 'group' is an analysis of identical markers).

☐ All plots are contour plots with outliers or pseudocolor plots.

☐ A numerical value for number of cells or percentage (with statistics) is provided.

Methodology

Sample preparation	*Not applicable*
Instrument	*Not applicable*
Software	*Not applicable*
Cell population abundance	*Not applicable*
Gating strategy	*Not applicable*

☐ Tick this box to confirm that a figure exemplifying the gating strategy is provided in the Supplementary Information.

Magnetic resonance imaging

Experimental design

Design type	*Not applicable*
Design specifications	*Not applicable*
Behavioral performance measures	*Not applicable*

Acquisition

Imaging type(s)	*Not applicable*
Field strength	*Not applicable*
Sequence & imaging parameters	*Not applicable*
Area of acquisition	*Not applicable*

Diffusion MRI ☐ Used ☐ Not used

Preprocessing

Preprocessing software	*Not applicable*
Normalization	*Not applicable*
Normalization template	*Not applicable*
Noise and artifact removal	*Not applicable*
Volume censoring	*Not applicable*

Statistical modeling & inference

Model type and settings	*Not applicable*
Effect(s) tested	*Not applicable*

Specify type of analysis: ☐ Whole brain ☐ ROI-based ☐ Both

Statistic type for inference (See Eklund et al. 2016)	*Not applicable*
Correction	*Not applicable*

Models & analysis

n/a	Involved in the study
☒	☐ Functional and/or effective connectivity
☒	☐ Graph analysis
☒	☐ Multivariate modeling or predictive analysis

Functional and/or effective connectivity	*Not applicable*
Graph analysis	*Not applicable*
Multivariate modeling and predictive analysis	*Not applicable*
